# Supplementary material for: Seven-month kinetics of SARS-CoV-2 antibodies and role of pre-existing antibodies to human coronaviruses
Source: Nat Commun. 2021 Aug 6;12:4740. doi: 10.1038/s41467-021-24979-9 (PMC8346582; doi:10.1038/s41467-021-24979-9)
Supplement: Supplementary file 1 — Supplementary Information [file 41467_2021_24979_MOESM1_ESM.pdf]

Seven-month kinetics of SARS-CoV-2 antibodies and role of pre-existing antibodies to human coronaviruses

Supplementary Information

		Total
Sex [a]	Male	161 (28%)
	Female	417 (72%)
Professional Category [a]	Nurse / Auxiliary / Stretcher-bearer	288 (50%)
	Physician	147 (25%)
	Lab technicians	45 (8%)
	Admin officers / Other[b]	98 (17%)
Age [c]		42.1 (11.6)
Daily contact with patients [a]	No	123 (21%)
	Yes	455 (79%)
Working in a COVID-19 unit [a]	No	315 (54%)
	Yes	263 (46%)
Close contact with COVID-19 confirmed or suspected case [a]	No	137 (24%)
	Yes	441 (76%)
Previously diagnosed of COVID-19 by RT-qPCR [a]	No	539 (93%)
	Yes	39 (7%)
Comorbidities [a,d]	No	517 (89%)
	Yes	61 (11%)
Household size [c]		2.8 (1.2)
Received Flu vaccine (2019-2020 season) [a]	No	339 (59%)
	Yes	239 (41%)
Reporting COVID-19 compatible symptoms within previous month [a]	No	368 (64%)
	Yes	210 (36%)

[a] n (Column percentage)
[b] Includes, cleaning, kitchen and maintenance staff
[c] Arithmetic Mean (SD) [n]
[d] Comorbidities include: heart and liver disease, diabetes, chronic respiratory and renal disease, cancers and autoimmune and other immunological disorders.

Supplementary Table 1. Baseline characteristics of study participants.

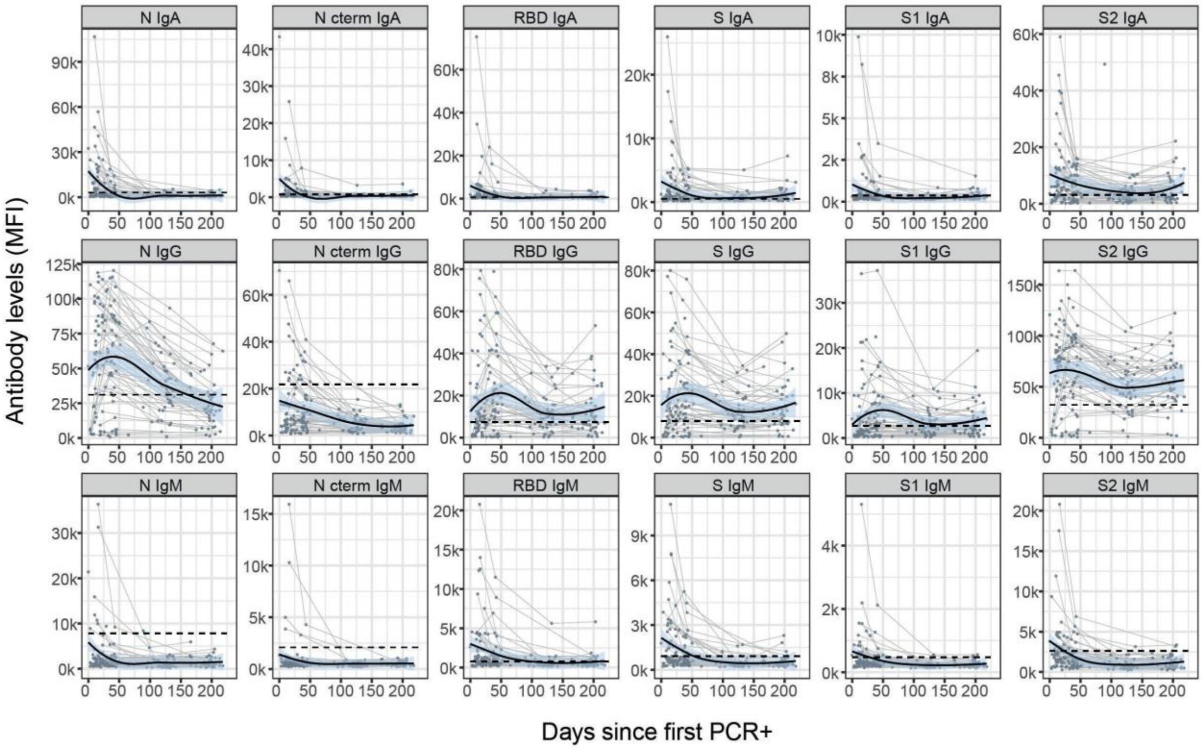

Supplementary Figure 1. **Kinetics of SARS-CoV-2 antibody levels since day of first positive rRT-PCR test.** Levels (median fluorescence intensity, MFI) of IgA, IgG and IgM against each antigen (Nucleocapsid full length protein (N), and its C-terminal domain, the Receptor Binding Domain (RBD), full S protein and its subregions S1 and S2). Data are shown only for the 67 participants who had a positive rRT-PCR. Up to four time points are shown per participant (paired samples joined by lines). The black solid line represents the fitted curve calculated using the LOESS (locally estimated scatterplot smoothing) method. Shaded areas represent 95% confidence intervals.

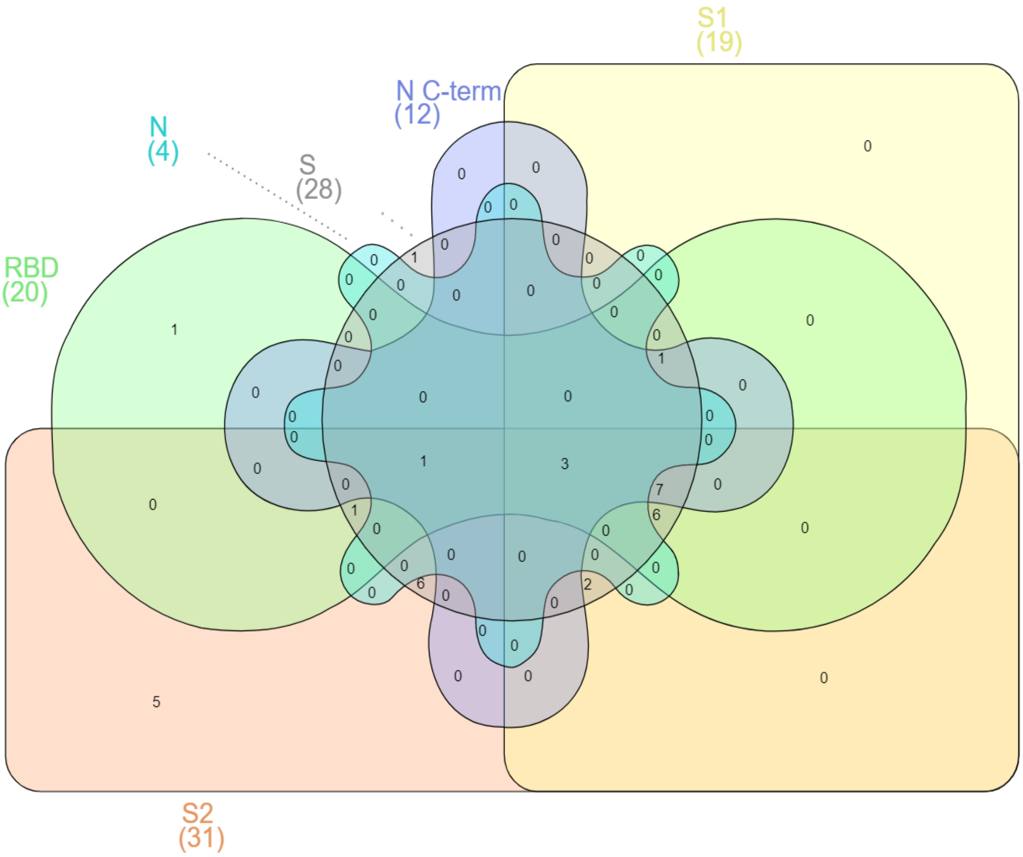

Supplementary Figure 2. Venn Diagram illustrating the overlap between antigen-specific IgGs in the “Sustainers/Increasers" group. Participants were grouped based on their antibody levels at M6 compared to the previous visit, individuals were labelled for each isotype-antigen pair as “Decayers” when the ratio of antibody levels between both visits was <1 and as “Sustainers/Increasers” when the ratio was ≥ 1. Here, we only represent participants who classify as “Sustainers/Increasers" for IgG against each of the studied antigens (n=34). The numbers of sustainers/increasers seropositive for IgG against the indicated antigen are between parentheses.

	Visit	All isotypes(%)<br>[95% CI]	IgA (%)<br>[95% CI]	IgG (%)<br>[95% CI]	IgM (%)<br>[95% CI]
Seroprevalence*	M0	78/578 (13.5%)<br>[10.8- 16.6%]	60/578 (10.4%)<br>[8.0-13.2%]	42/578 (7.6%)<br>[5.3-9.7%]	53/578 (9.2%)<br>[6.9-11.8%]
	M1	86/566 (15.6%)<br>[12.3-18.4%]	61/566 (10.8%)<br>[8.3-13.6%]	59/566 (10.8%)<br>[8.0-13.2%]	54/566 (9.6%)<br>[7.2-12.3%]
	M6	83/507 (16.4%)<br>[13.3-19.9%]	58/507 (11.4%)<br>[8.8-14.5%]	58/507 (11.4%)<br>[8.8-14.5%]	35/507 (6.9%)<br>[4.9-9.5%]
Seroconversion*	M1	23/500 (4.6%)	20/500 (4.0%)	21/500 (4.2%)	19/500 (3.8%)
	M6	13/478 (2.7%)	10/478 (2.1%)	12/478 (2.5%)	11/478 (2.3%)
Seroreversion	M1	7/78 (9.0%)	18/60 (30.0%)	2/44 (4.5%)	13/53 (24.5%)
	M3	6/88 (6.8%)	14/61 (23.0%)	6/61 (9.8%)	16/54 (29.6%)
	M6	0/49 (0.0%)	1/30 (3.3%)	1/44 (2.3%)	1/12 (8.3%)

Supplementary Table 2. Seroprevalence, seroconversion and seroreversion rates for each visit.

*Only participants with previous evidence of SARS-CoV-2 infection were invited to visit M3, thus, no seroprevalence or seroconversion data are presented for this visit.

	Seropositive participants up to M6 (%) n= 110	Seronegative participants up to M6 (%) n= 468	OR (95% CI)	
			Univariable Logistic Regression	p-value
Age	42.2	42.1	1.00 (0.98-1.02)	0.9365[d]
Sex				
Females	84 (76%)	334 (71%)	1.33 (0.83-2.20)	0.241[e]
Males	26 (24%)	134 (29%)	1.0 (Ref)	
Job function				
Nurses and auxiliary health professionals [a]	63 (57%)	225 (48%)	1.0 (Ref)	
Laboratory and other technicians	9 (8%)	35 (7%)	0.92 (0.40-1.94)	0.8314[e]
Physicians and psychologists	18 (16%)	127 (27%)	0.49 (0.27-0.85)	0.0138 *[e]
Others [b]	20 (18%)	81 (17%)	0.88 (0.49-1.53)	0.6618[e]
Involved in clinical care				
Yes	89 (81%)	366 (78%)	1.17 (0.70-2.01)	0.56[e]
No	21 (19%)	102 (22%)	1.0 (Ref)	
Worked in a COVID ward				
Yes	67 (61%)	299 (64%)	0.90 (0.59-1.39)	0.634[e]
No	43 (39%)	169 (36%)	1.0 (Ref)	
Baseline illness [c]				
Yes	19 (17%)	99 (21%)	0.79 (0.45-1.33)	0.385[e]
No	91 (83%)	369 (79%)	1.0 (Ref)	
Chronic medication				
Yes	18 (16%)	101 (22%)	0.69 (0.39-1.18)	0.19[e]
No	92 (84%)	364 (77%)	1.0 (Ref)	
Symptomatic				
Yes	73 (66%)	6 (1%)	153.2 (67.3-416.3)	<2e-16 ***[e]
No	37 (33%)	462 (99%)	1.0 (Ref)	
Nº children co-living	0.49	0.47	1.02 (0.78-1.30)	0.901[d]
Nº people household	2.79	2.74	1.04 (0.87-1.24)	0.679[d]
**Smoker**				
Yes	20 (18%)	104 (22%)	0.78 (0.45-1.31)	0.373[e]
No	87 (79%)	355 (76%)	1.0 (Ref)	

Supplementary Table 3. Univariable analysis of factors associated with having detectable SARS-CoV-2 antibodies at M6 (IgM and/or IgG and/or IgA against each antigen (Nucleocapsid (N), and its C-terminal domain, the Receptor Binding Domain (RBD), full S protein and its subregions S1 and S2). All factors explored are included in the table. OR: Odds Ratio.

* p-value <0.05
*** p-value < 0.001
[a] Includes stretcher-bearer.
[b] Includes, cleaning, kitchen and maintenance staff
[c] Comorbidities include heart and liver disease, diabetes, chronic respiratory and renal disease, cancers and autoimmune, and other immunological disorders.
[d] T-test (two-sided)
[e] Chi-squared test

	Dependent variable: *Neutralizing capacity (%)*
	**Beta (SE)**
Component 1[a]	3.893*** (0.482)
Component 2	0.932 (0.649)
Component 3	-0.923 (0.811)
Component 4	-0.585 (0.918)
Component 5 [b]	3.579*** (1.108)
Constant	34.488*** (1.151)
Observations	56
R^2	0.614
Adjusted R^2	0.575
Residual Std. Error	8.615 (df = 50)
F Statistic	15.903*** (df = 5; 50)
Note:	$^{*}p^{<0.1; >**}p^{<0.05; >***}p^{<0.01>}$

[a] Mostly contributing to the Component 1: S1 IgG (0.374) / S IgG (0.381)

[b] Mostly contributing to the Component 5: N C terminal IgG (-0.656) / S2 IgM (0.355)

Supplementary Table 4. Principal Components Regression. Linear regression analysis of the neutralization capacity (%) employing principal components analysis in place of the original isotype-antigen variables. SE: Standard Error.

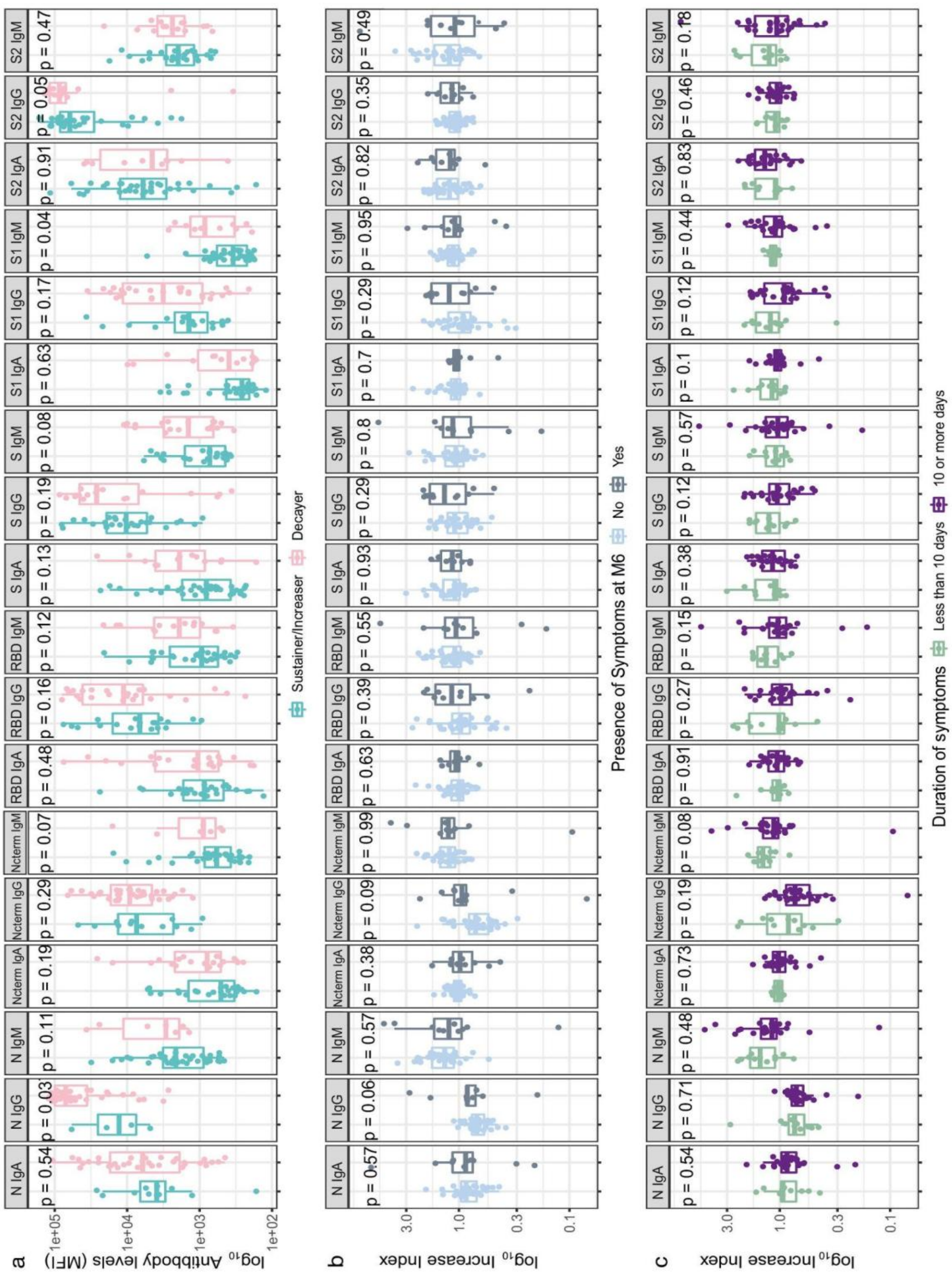

Supplementary Figure 3. Comparisons of serological and clinical characteristics between sustainers/increasers and decayers. **a)** Differences in antibody levels at seroconversion between sustainers/increasers and decayers (median fluorescence intensity, MFI) of IgA, IgG and IgM against each antigen (Nucleocapsid (N), and its C-terminal domain, the Receptor Binding Domain (RBD), full S protein and its subregions S1 and S2)) (n = 110). **b)** Differences in antibody increase index (represented in log scale) between seropositive participants who reported symptoms and those who did not in month 6 (M6) after recovering from a previous SARS-CoV-2 infection (n=53) **c)** Differences in antibody increase index (represented in log scale) between seropositive

symptomatic participants who reported less than 10 days of symptom duration and those who reported more than 10 days (n=53). The center line of boxes depicts the median; the lower and upper hinges correspond to the first and third quartiles; the distance between the first and third quartiles corresponds to the interquartile range (IQR); whiskers extend from the hinge to the highest or lowest value within 1.5 × IQR of the respective hinge. Wilcoxon rank test was used to assess statistically significant differences in antibody levels between groups**.**

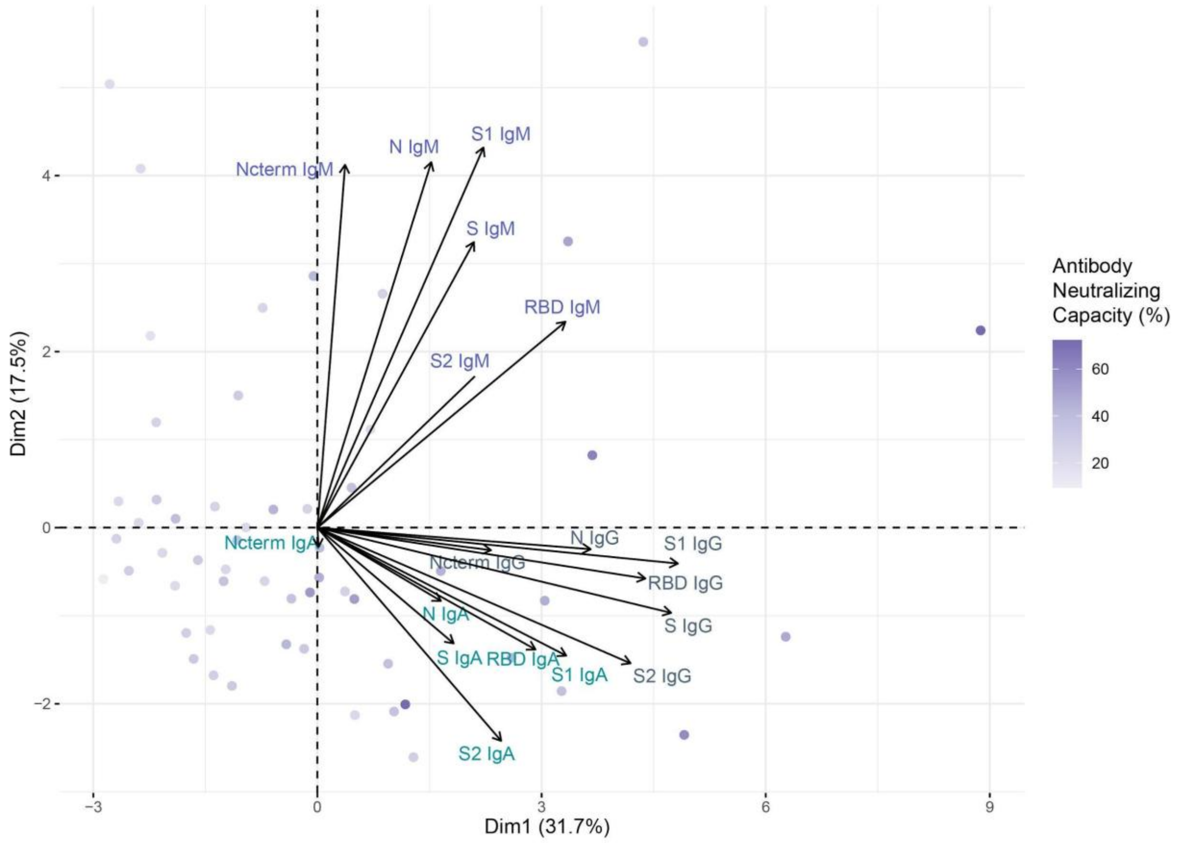

Supplementary Figure 4. Principal Components Analysis (PCA) Biplot. Vectors represent all antigen/isotype pairs and their contribution to the variance in the antibody neutralization percentage represented in the two main dimensions axes. Observations are plotted according to their neutralization percentage at fourth visit (M6).

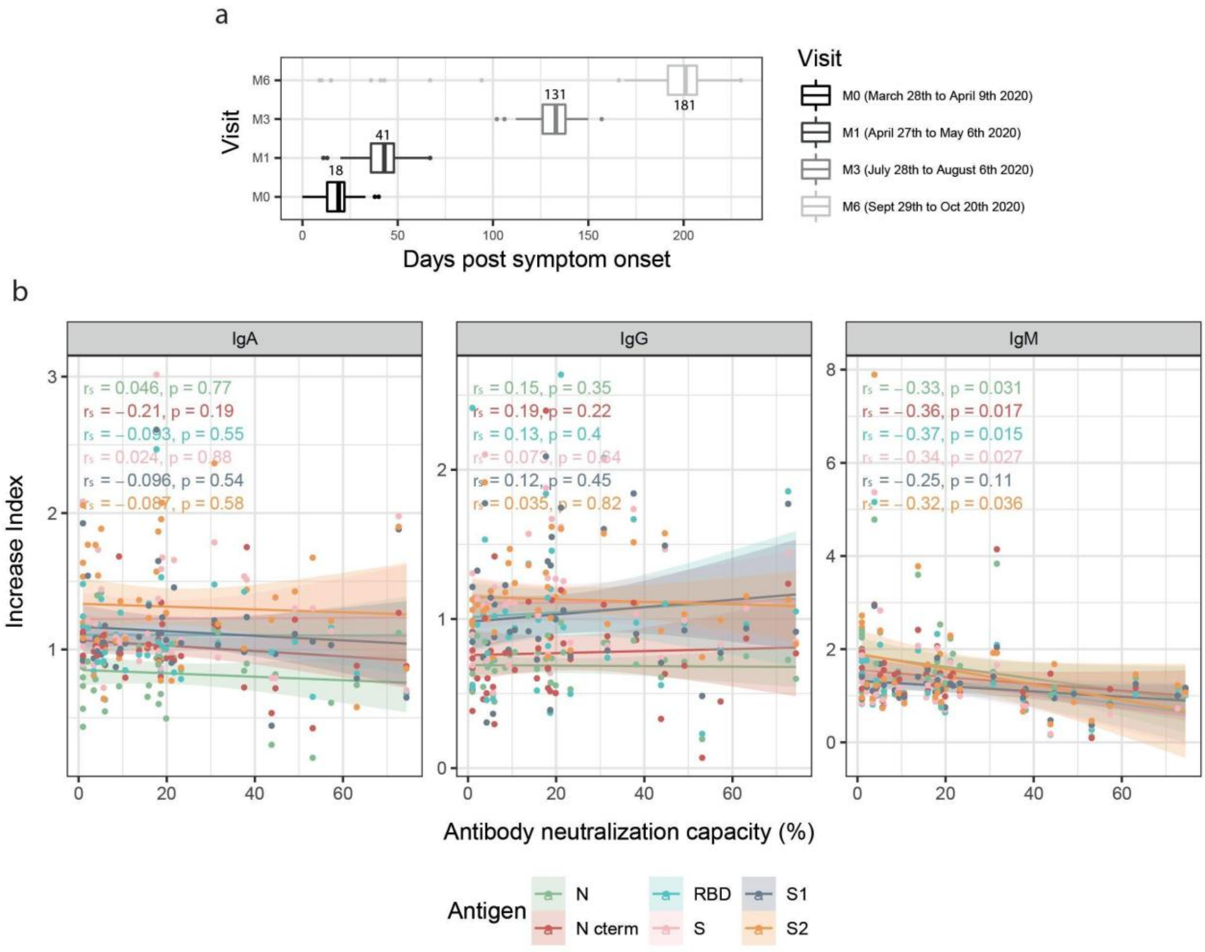

Supplementary Figure 5. a) Sample collection timeline.The center line of boxes depicts the median of days post symptom onset; the lower and upper hinges correspond to the first and third quartiles; the distance between the first and third quartiles corresponds to the interquartile range (IQR); whiskers extend from the hinge to the highest or lowest value within 1.5 × IQR of the respective hinge. **b)** Correlations between antibody increase index and neutralization capacity. Spearman's rank correlation test between antibody increase index (MFI increase between M6 and previous visit) of IgA, IgG and IgM against each study antigen (Nucleocapsid full length protein (N), and its C-terminal domain, the Receptor Binding Domain (RBD), full S protein and its subregions S1 and S2), and the plasma neutralization capacity at M0 (as a percentage of RBD-ACE2 binding inhibition). Spearman test was used to calculate the p-values and r_s correlation coefficients are color-coded for each antigen/isotype pair. Colored lines represent the fitted curve calculated using the linear model method. Shaded areas represent 95% confidence intervals (n=43).

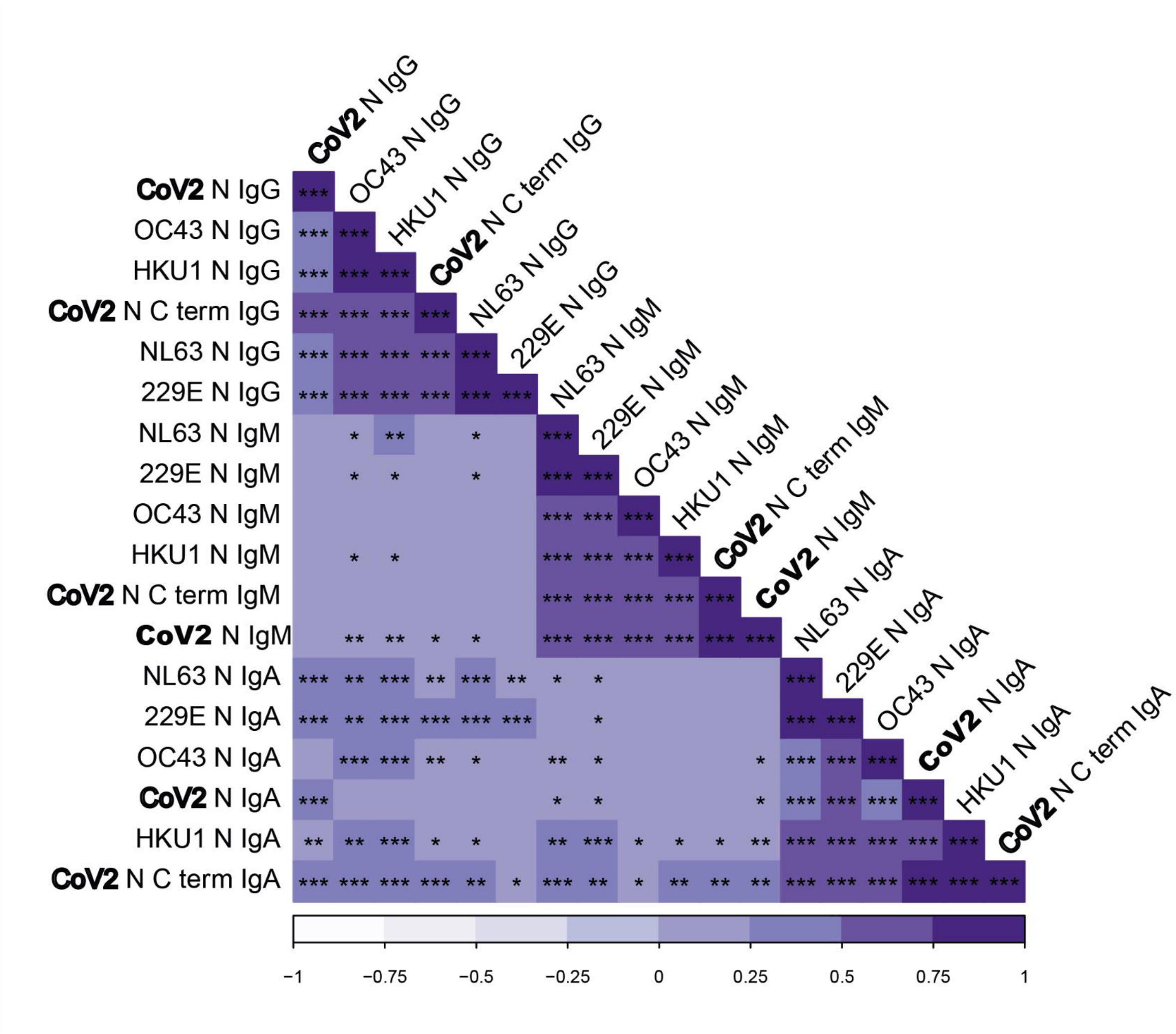

Supplementary Figure 6. Correlation plot with hierarchical clustering showing the correlations between IgG, IgM and IgA levels against Nucleocapsid (N) protein from the four seasonal human coronaviruses (HCoV) and SARS-CoV-2 in 101 pre-pandemic samples. Spearman test was used to calculate the p-values and r_s correlation coefficients. Significance level in the correlations is represented by * ($p < 0.05$), ** ($p < 0.01$), *** ($p < 0.001$). Bold letters highlight SARS-CoV-2 coronavirus for visualization purposes.

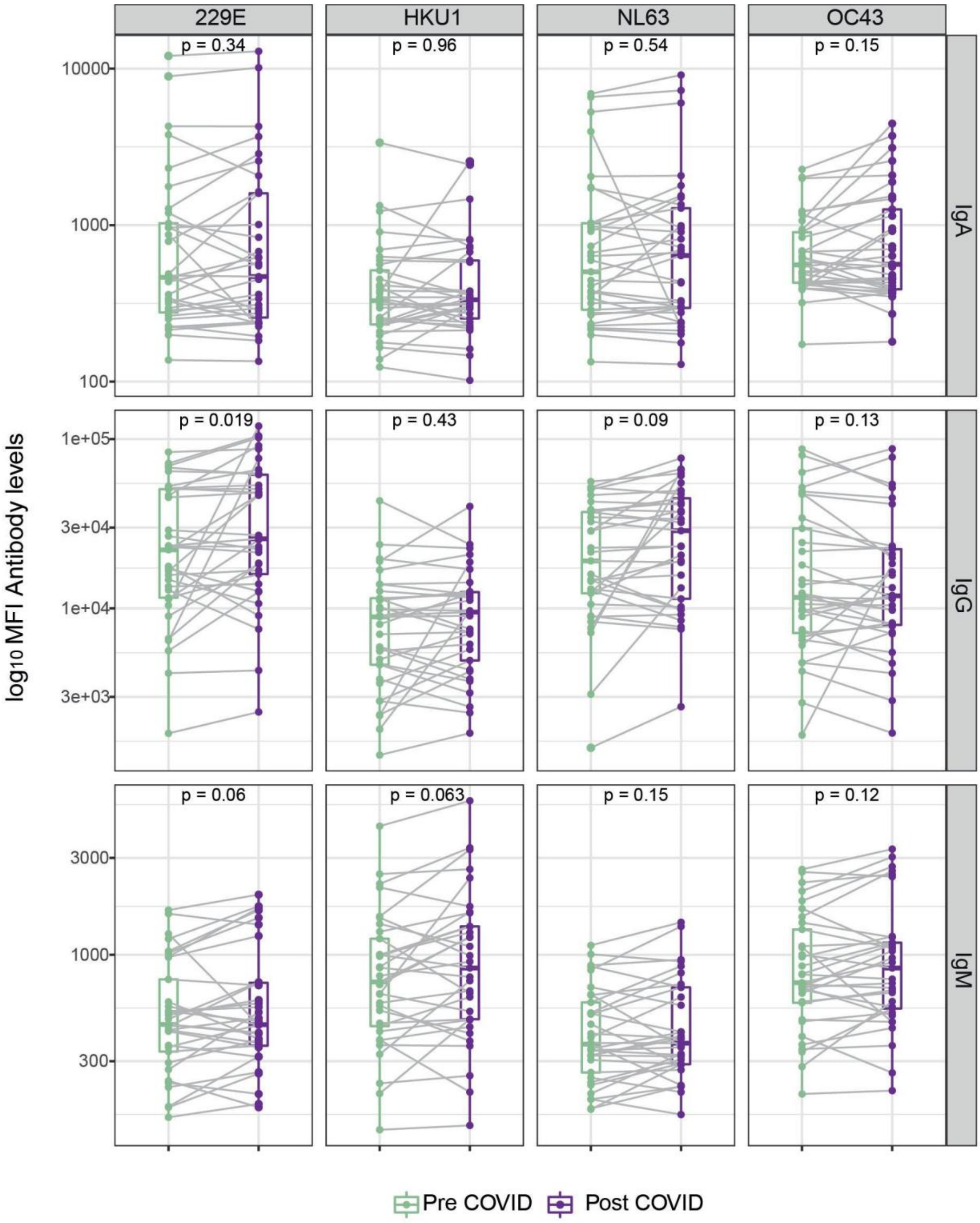

Supplementary Figure 7. Boxplots comparing the levels (median fluorescence intensity, MFI) of IgA, IgG and IgM against the Nucleocapsid (N) protein of the four seasonal human coronaviruses (HCoV) before and after SARS-CoV-2 seroconversion (n = 33). The center line of boxes depicts the median; the lower and upper hinges correspond to the first and third quartiles; the distance between the first and third quartiles corresponds to the interquartile range (IQR); whiskers extend from the hinge to the highest or lowest value within 1.5 × IQR of the respective hinge. Paired T-test

(two-sided) was used to assess statistically significant differences in antibody levels between groups.

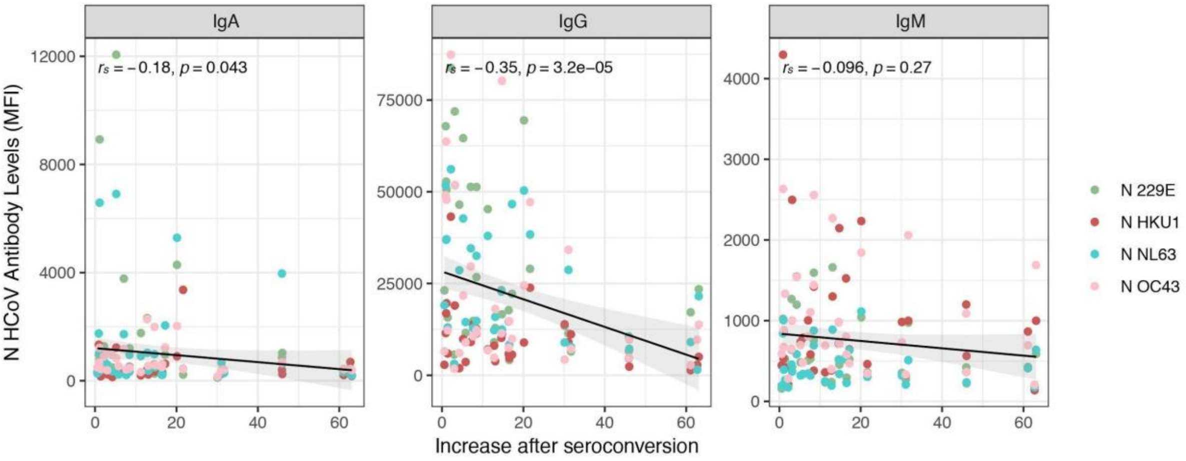

Supplementary Figure 8. Linear regression models showing the relation between antibody levels against the four HCoV N proteins (three isotypes added) and the anti-SARS-CoV-2 N antibody ratio of seroconversion (seroconversion levels/baseline levels) for all three isotypes. Spearman test was used to calculate the p-values and r_s correlation coefficients are given for each isotype. Black lines represent the fitted curve calculated using the linear model method. Shaded areas represent 95% confidence intervals (n = 33).

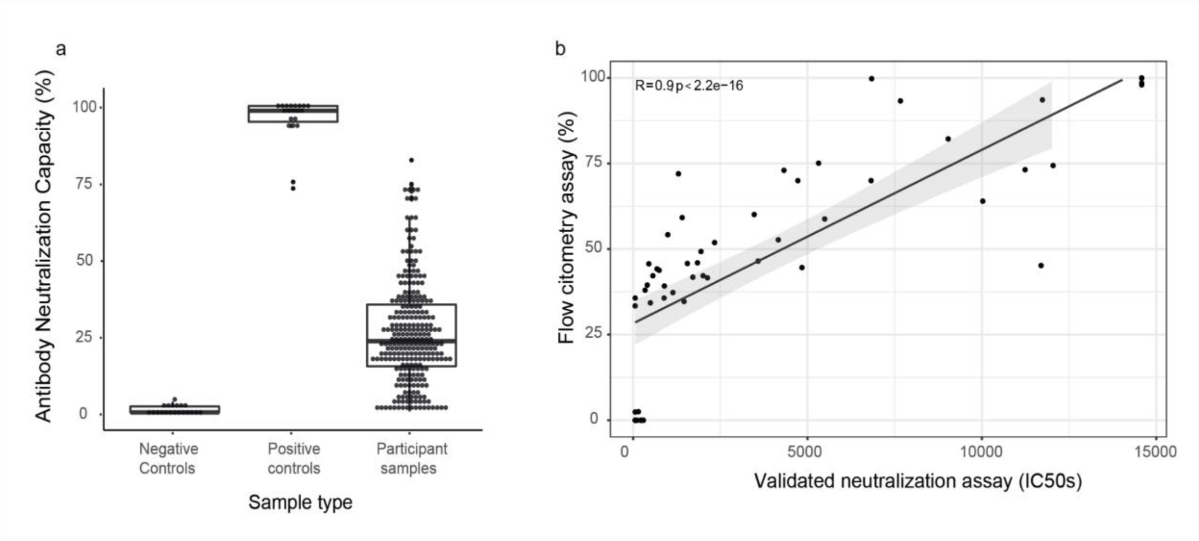

Supplementary Figure 9. a) Antibody neutralization capacity is compared between negative controls (n = 20), positive controls (n = 20) and participants (n = 578). The center line of boxes depicts the median antibody neutralization capacity; the lower and upper hinges correspond to the first and third quartiles; the distance between the first and third quartiles corresponds to the interquartile range (IQR); whiskers extend from the hinge to the highest or lowest value within 1.5 × IQR of the respective hinge. b) Correlation between neutralization values as per the flow cytometry assay used for this work and values from a cross-validated pseudovirus neutralization assay (ID50 - half maximal dilutions concentrations) (n = 55) (R = 0.9, p-value = 2.2 x 10^{-16}). Spearman test was used to calculate the p-value and R correlation coefficient. Shaded areas represent 95% confidence intervals.

Supplementary Methods

Quantification of antibodies to SARS-CoV-2 by Luminex

The levels of IgG, IgM and IgA were assessed in single replicates by high-throughput multiplex quantitative suspension array technology (qSAT). The assay was performed in 6 plates of 384 wells with samples from the same individual in the same plate (samples from visit M0 to M6).The SARS-CoV-2 antigens included were the spike full protein (S) (aa 1-1213 expressed in Expi293 and His tag-purified), the S1 (aa 1-681, expressed in Expi293 and His tag-purified) and S2 (purchased from SinoBiologicals), the receptor binding domain (RBD) (fused with C-terminal 6xHis and StrepTag purification sequences and purified from supernatant of lentiviral-transduced CHO-S cells cultured under a fed-batch system), the nucleocapsid full protein (N) and the specific N C-terminal region, and the four HCoV N full length proteins (expressed in *E. coli* and His tag-purified). Assay performance was previously established as 100% specificity and 95.78% sensitivity for seropositivity 14 days after symptoms onset [1].

Coupling of proteins to microspheres

MagPlex® polystyrene 6.5 µm COOH-microspheres (Luminex Corp, Austin, TX, USA) were washed, sonicated and activated with Sulfo-NHS (*N*-hydroxysulfosuccinimide) and EDC (1-Ethyl-3-[3-dimethylaminopropyl]carbodiimide hydrochloride) (Thermo Fisher Scientific Inc.,Waltham USA). Next, microspheres were washed and resuspended in 50 mM MES pH 5.0 (MilliporeSigma, St. Louis, USA). The recombinant proteins were then incubated with the microspheres at the optimal concentrations (from 10 to 50 µg/mL) and left at 4°C on a shaker overnight. Coupled microspheres were resuspended in PBS with 1% BSA to covalently block the free carboxylic group (-COOH) absorbing most of the non-specific binding to secondary or tertiary antibodies during assay steps [2] and heterophilic antibody binding seen in previous systems [3]. Microspheres recovery were quantified on a Guava® easyCyte™ Flow Cytometer (Luminex Corporation, Austin, USA). Equal amounts of each antigen-coupled microspheres were multiplexed and stored at 2000 microspheres/µL at 4°C, protected from light.

qSAT assay

Antigen-coupled microspheres were added to a 384-well µClear® flat bottom plate (Greiner Bio-One, Frickenhausen, Germany) in multiplex (2000 microspheres per analyte per well) in a volume of 90 µL of Luminex Buffer (1% BSA, 0.05% Tween 20, 0.05% sodium azide in PBS) using 384 channels Integra Viaflo semi-automatic device (96/384, 384 channel pipette). Two hyperimmune pools (one for IgG and IgA, and another one for IgM) were used as positive controls in each plate assay for QA/QC purposes and were prepared at 2-fold, 8 serial dilutions from 1:12.5. Pre-pandemic samples were used as negative controls to estimate the cut off of seropositivity. Ten µL of each dilution of the positive control, negative controls and test samples (prediluted 1:50 in 96 round-bottom well plates), were added to a 384-well plate using Assist Plus Integra device with 12 channels Voyager pipette (final test sample dilution of 1:500). To quantify IgM responses, test samples and controls were pre-treated with anti-Human

IgG (Gullsorb) at 1:10 dilution, to avoid IgG interferences. Technical blanks consisting of Luminex Buffer and microspheres without samples were added in 4 wells to detect and adjust for non-specific microsphere signals. Plates were incubated for 1 h at room temperature in agitation (Titramax 1000) at 900 rpm and protected from light. Then, the plates were washed three times with 200 µL/well of PBS-T (0.05% Tween 20 in PBS), using BioTek 405 TS (384-well format). Twenty five µL of goat anti-human IgG-phycoerythrin (PE) (GTIG-001, Moss Bio) diluted 1:400, goat anti-human IgA-PE (GTIA-001, Moss Bio) 1:200, or goat anti-human IgM-PE (GTIM-001, Moss Bio) 1:200 in Luminex buffer were added to each well and incubated for 30 min. Plates were washed and microspheres resuspended with 80 µL of Luminex Buffer, covered with an adhesive film and sonicated 20 seconds on sonicator bath platform, before acquisition on the Flexmap 3D® reader. At least 50 microspheres per analyte per well were acquired, and median fluorescence intensity (MFI) was reported for each analyte.

Neutralizing antibodies

The stable cell line 300.19-ACE2 was obtained by transfecting 300.19 cells with a plasmid encoding human ACE2 cDNA (SinoBiological) with an Amaxa cell line Nucleofector kit V, followed by hygromycin selection and subsequent subcloning. RBD-mFc fusion protein, containing RBD fused to the Fc region of murine IgG1 was obtained by cloning RBD amplified from the pcDNA3-SARS-CoV-2-S-RBD-Fc (Addgene) into the PFUSE-mIGg1-Fc1 (InvivoGen). HEK-293T cells were transiently transfected with the RBD-mFc plasmid using polyethylenimine as previously described [4]. The supernatant containing the RBD-mFc protein was collected 7 days after transfection, and concentrated 4-fold using an Amicon Ultra-15 Centrifugal Filter Unit with an Ultracel-30 membrane (Millipore).
A total of 1.2 x10^3 300.19-ACE2 cells per well in a 96-well plate were incubated for 30 min at 4°C with 4 mg/mL of RBD-mFc fusion protein previously exposed to diluted plasma (1:50) for 30 min at 4°C. Samples were stained with anti-mouse IgG-PE 1:200 (Jackson ImmunoResearch), washed, and analyzed by Flow cytometry using standard procedures. Samples were acquired with a FACSCanto II (BD Biosciences) and analyzed with FlowJo Xv10.0.7 (Tree Star, Inc) software [4].
Pseudovirus-based neutralization assay using HIV-based pseudovirus and ACE2 expressing 293T cells is described in Pradenas et al. [5]. This assay has been validated by direct comparison of IC50 neutralization values obtained using pseudoviruses infecting ACE2 expressing 293 cells and replicative viruses infecting Vero cells in Trinité et al. [6].

Supplementary References

[1] Dobaño, C. et al. Highly sensitive and specific multiplex antibody assays to quantify immunoglobulins M, A and G against SARS-CoV-2 antigens. (2020). Preprint at 10.1101/2020.06.11.147363

[2] Waterboer T, Sehr P, Pawlita M. Suppression of non-specific binding in serological Luminex assays. *J Immunol Methods* **309**, 200–4 (2006)

[3] Martins TB, Pasi BM, Litwin CM, Hill HR. Heterophile antibody interference in a multiplexed fluorescent microsphere immunoassay for quantitation of cytokines in human serum. *Clin Diagn Lab Immunol* **11**, 325-9 (2004)

[4] Martínez-Vicente P, Farre D, Sánchez C, Alcamí A, Engel P, Angulo A. Subversion of natural killer cell responses by a cytomegalovirus-encoded soluble CD48 decoy receptor. *PLoS Pathog* **15** (2019)

[5] Pradenas E. et al. Stable neutralizing antibody levels six months after mild and severe COVID-19 episode. Med 3, 313-320 (2021)

[6] Trinité, B., et al. SARS-CoV-2 infection elicits a rapid neutralizing antibody response that correlates with disease severity. Sci Rep 11, 2608 (2021).
