# Supplementary material for: Seven-month kinetics of SARS-CoV-2 antibodies and role of pre-existing antibodies to human coronaviruses
Source: Nat Commun. 2021 Aug 6;12:4740. doi: 10.1038/s41467-021-24979-9 (PMC8346582; doi:10.1038/s41467-021-24979-9)
Supplement: Supplementary file 3 — Reporting Summary [file 41467_2021_24979_MOESM3_ESM.pdf]

nature portfolio

Corresponding author(s): Gemma Moncunill and Carlota Dobaño

Last updated by author(s): July 14, 2021

Reporting Summary

Nature Portfolio wishes to improve the reproducibility of the work that we publish. This form provides structure for consistency and transparency in reporting. For further information on Nature Portfolio policies, see our Editorial Policies and the Editorial Policy Checklist.

Statistics

For all statistical analyses, confirm that the following items are present in the figure legend, table legend, main text, or Methods section.

n/a	Confirmed	
☐	☒	The exact sample size (*n*) for each experimental group/condition, given as a discrete number and unit of measurement
☐	☒	A statement on whether measurements were taken from distinct samples or whether the same sample was measured repeatedly
☐	☒	The statistical test(s) used AND whether they are one- or two-sided<br>*Only common tests should be described solely by name; describe more complex techniques in the Methods section.*
☐	☒	A description of all covariates tested
☐	☒	A description of any assumptions or corrections, such as tests of normality and adjustment for multiple comparisons
☐	☒	A full description of the statistical parameters including central tendency (e.g. means) or other basic estimates (e.g. regression coefficient) AND variation (e.g. standard deviation) or associated estimates of uncertainty (e.g. confidence intervals)
☐	☒	For null hypothesis testing, the test statistic (e.g. *F*, *t*, *r*) with confidence intervals, effect sizes, degrees of freedom and *P* value noted<br>*Give P values as exact values whenever suitable.*
☒	☐	For Bayesian analysis, information on the choice of priors and Markov chain Monte Carlo settings
☒	☐	For hierarchical and complex designs, identification of the appropriate level for tests and full reporting of outcomes
☐	☒	Estimates of effect sizes (e.g. Cohen's *d*, Pearson's *r*), indicating how they were calculated

Our web collection on statistics for biologists contains articles on many of the points above.

Software and code

Policy information about availability of computer code

Data collection	We filled out a standardized electronic questionnaire programmed in Research electronic data capture (REDCap version 8.8.2) from each participant.
Data analysis	Data analysis was carried out using R version 4.0.3 (packages tidyverse, corrplot, FactormireR, pls and MASS) and FlowJoXv 10.0.7 (TReeStar, Inc.)

For manuscripts utilizing custom algorithms or software that are central to the research but not yet described in published literature, software must be made available to editors and reviewers. We strongly encourage code deposition in a community repository (e.g. GitHub). See the Nature Portfolio guidelines for submitting code & software for further information.

Data

Policy information about availability of data

All manuscripts must include a data availability statement. This statement should provide the following information, where applicable:

- Accession codes, unique identifiers, or web links for publicly available datasets
- A description of any restrictions on data availability
- For clinical datasets or third party data, please ensure that the statement adheres to our policy

Anonymized data used for this analysis will be available upon manuscript acceptance and made public under the title of this publication at https://doi.org/10.34810/data125.

March 2021

Field-specific reporting

Please select the one below that is the best fit for your research. If you are not sure, read the appropriate sections before making your selection.

[x] Life sciences [ ] Behavioural & social sciences [ ] Ecological, evolutionary & environmental sciences

For a reference copy of the document with all sections, see nature.com/documents/nr-reporting-summary-flat.pdf

Life sciences study design

All studies must disclose on these points even when the disclosure is negative.

Sample size	A 570 sample size was determined to assess the seroprevalence against SARS-CoV-2 with a precision of 5% and a 95% CI, a loss to follow up between month 0 and month 1 of 5% and assuming that the prevalence at month 0 was 30% and at month 1 of 50% with a finite population, 578 health care workers were recruited.<br>If we will include 379 experimental units, we will have an accuracy of 5.03% in estimating a proportion using an asymptotic Normal CI at 95% bilateral, assuming that the real proportion is 50% (the worst case scenario) at point time one month after the recruitment.<br>We expect a 30% positive proportion of COVID19 at recruitment and a 5% of lost to follow up between two time points (recruitment and month one). Then, we estimated that 570 participants is the pertinent sample size at recruitment to have enough statistical power to detect a 50% of positive COVID19 prevalence at time Month 1 (M1) after the recruitment with a 5% accuracy. We have calculated the sample size assuming a finite population.
Data exclusions	We excluded 5 recruited participants after re-checking inclusion and exclusion criteria. These 5 participants were interviewed at baseline but they had never been selected in the random sample, so we excluded them from the final analysis.
Replication	We did all analysis using ad-hoc p R files programmed by the researchers
Randomization	We stratified the sample by age (less 45 years old and higher or equal 45 years) to ensure the same proportion of population in the sample research. We approached the first 1172 people following the order of the study random list. One for each group of age. We tested all samples in the neutralisation assays, so we did not require any additional randomization of the samples.
Blinding	Blinding was not performed since the purpose of this work was not experimental.

Reporting for specific materials, systems and methods

We require information from authors about some types of materials, experimental systems and methods used in many studies. Here, indicate whether each material, system or method listed is relevant to your study. If you are not sure if a list item applies to your research, read the appropriate section before selecting a response.

Materials & experimental systems

n/a	Involved in the study	
[ ]	[x]	Antibodies
[ ]	[x]	Eukaryotic cell lines
[x]	[ ]	Palaeontology and archaeology
[x]	[ ]	Animals and other organisms
[ ]	[x]	Human research participants
[x]	[ ]	Clinical data
[x]	[ ]	Dual use research of concern

Methods

n/a	Involved in the study	
[x]	[ ]	ChIP-seq
[ ]	[x]	Flow cytometry
[x]	[ ]	MRI-based neuroimaging

Antibodies

Antibodies used	Goat anti-human IgG-phycoerythrin (PE) (GTIG-001, Moss Bio)<br>Goat anti-human IgA-PE (GTIA-001, Moss Bio)<br>Goat anti-human IgM-PE (GTIM-001, Moss Bio)<br>Goat anti-mouse IgG-PE (1151160711, Jackson ImmunoResearch)
Validation	Assay performance was previously established as 100% specificity and 95.78% sensitivity for seropositivity 14 days after symptoms onset (Dobaño C, et al. J Clin Microbiol 2020:JCM.01731-20.)

Eukaryotic cell lines

Policy information about cell lines

Cell line source(s)	The stable cell line 300.19-ACE2 was generated by transfecting 300.19 cells with a plasmid encoding human ACE2 cDNA (SinoBiological) with an Amaxa cell line Nucleofector kit V, followed by hygromycin selection and subsequent subcloning. The [illegible]

	300.19 cell line was obtained from F.W. Alt (Harvard Medical School, Boston, MA, USA), and described in Reth et al., 1986, EMBO J. 5:2131-2138.<br>HEK-293T cells (ATCC CRL-1573).
Authentication	The 300.19 cell line (a murine pre-B cell line) was authenticated by Flow cytometry Immunophenotyping (CD19+, CD21+/-, CD22-, IgM-, IgG-, IgD-, B220-, CD3-). HEK-293T cells were not authenticated.
Mycoplasma contamination	All cell lines tested negative for mycoplasma contamination.
Commonly misidentified lines<br>(See ICLAC register)	No commonly misidentified cell lines were used in the study.

Human research participants

Policy information about studies involving human research participants

Population characteristics	At baseline, the mean age of participants was 42.1 years (SD: 11.6) and 72.1% were females and around half (288/578, 49.8%) were nurses, auxiliary nurses or stretcher-bearers, and 25.4% (147/578) were physicians. From the initial cohort, 507 individuals participated in a fourth visit six months after baseline, mean age 42.7 (SD 11.2) and 72% female. The study was conducted in healthy participants (11% had comorbidities at baseline).
Recruitment	Participants were selected on a random sample basis. From a total number of 5598 healthcare workers registered at Hospital Clínic de Barcelona as of March 9 th , 2020, the first 1172 randomly selected individuals were approached, following the list order. We had a high response rate, but self selection bias could have influenced our results as during the first COVID19 peak and lockdown, there was a lot of healthcare pressure in COVID19 wards and workers that were doing telework were not able to come as they did not want to expose themselves. See García-Basteiro et al. Nat Comms (2020) for more details.
Ethics oversight	The study was approved by the Ethics Committee at HCB (Ref number: HCB/2020/0336)

Note that full information on the approval of the study protocol must also be provided in the manuscript.

Flow Cytometry

Plots

Confirm that:

☐ The axis labels state the marker and fluorochrome used (e.g. CD4-FITC).

☐ The axis scales are clearly visible. Include numbers along axes only for bottom left plot of group (a 'group' is an analysis of identical markers).

☐ All plots are contour plots with outliers or pseudocolor plots.

☐ A numerical value for number of cells or percentage (with statistics) is provided.

Methodology

Sample preparation	RBD-mFc fusion protein, containing RBD fused to the Fc region of murine IgG1 was obtained by cloning RBD amplified from the pcDNA3-SARS-CoV-2-S-RBD-Fc (Addgene) into the PFUSE-mIGg1-Fc1 (InvivoGen). HEK-293T cells were transiently transfected with the RBD-mFc plasmid using polyethylenimine as previously described [AR5]. The supernatant containing the RBD-mFc protein was collected 7 days after transfection, and concentrated 4-fold using an Amicon Ultra-15 Centrifugal Filter Unit with an Ultracel-30 membrane (Millipore).<br>A total of 1.2 x103 300.19-ACE2 cells per well in a 96-well plate were incubated for 30 min at 4°C with 4 mg/mL of RBD-mFc fusion protein previously exposed to diluted plasma (1:50) for 30 min at 4°C. Samples were stained with anti-mouse IgG-PE 1:200(Jackson ImmunoResearch), washed, and analyzed by Flow cytometry using standard procedures.
Instrument	FACSCanto II (BD Biosciences)
Software	FlowJo Xv10.0.7 (Tree Star, Inc) software
Cell population abundance	100%. 300.19-ACE2 cells - It is a cell line
Gating strategy	N/A FSC/SSC gating on live cells. Gating does not apply because it is a FACS on a cell line (and not on a cell subset).

☐ Tick this box to confirm that a figure exemplifying the gating strategy is provided in the Supplementary Information.
